# High dietary salt does not significantly affect plasma 25-hydroxyvitamin D concentrations of Sprague Dawley rats

**DOI:** 10.1186/1756-0500-3-332

**Published:** 2010-12-09

**Authors:** Myrtle Thierry-Palmer, Teclemicael K Tewolde, Neremiah L Emmett, Mohamed A Bayorh

**Affiliations:** 1 Department of Microbiology, Biochemistry, and Immunology, Morehouse School of Medicine, 720 Westview Dr. S.W., Atlanta, Georgia 30310-1495, USA; 2 Department of Physiology, Morehouse School of Medicine, 720 Westview Dr. S.W., Atlanta, Georgia 30310-1495, USA; 3 Department of Pharmacology and Toxicology, Morehouse School of Medicine, 720 Westview Dr. S.W., Atlanta, Georgia 30310-1495, USA

## Abstract

**Background:**

The Dahl salt-sensitive rat, but not the Dahl salt-resistant rat, develops hypertension and hypovitaminosis D when fed a high salt diet. Since the salt-sensitive rat and salt-resistant rat were bred from the Sprague Dawley rat, the aim of this research was to test the hypothesis that salt-resistant and Sprague Dawley rats would be similar in their vitamin D endocrine system response to high salt intake.

**Findings:**

Sprague Dawley, salt-sensitive, and salt-resistant rats were fed high (80 g/kg, 8%) or low (3 g/kg, 3%) salt diets for three weeks. The blood pressure of Sprague Dawley rats increased from baseline to week 3 during both high and low salt intake and the mean blood pressure at week 3 of high salt intake was higher than that at week 3 of low salt intake (*P *< 0.05). Mean plasma 25-hydroxyvitamin D concentrations (marker of vitamin D status) of Sprague Dawley, salt-sensitive, and salt-resistant rats were similar at week 3 of low salt intake. Mean plasma 25-hydroxyvitamin D concentrations of Sprague Dawley and salt-resistant rats were unaffected by high salt intake, whereas the mean plasma 25-hydroxyvitamin D concentration of salt-sensitive rats at week 3 of high salt intake was only 20% of that at week 3 of low salt intake.

**Conclusions:**

These data indicate that the effect of high salt intake on the vitamin D endocrine system of Sprague Dawley rats at week 3 was similar to that of salt-resistant rats. The salt-sensitive rat, thus, appears to be a more appropriate model than the Sprague Dawley rat for assessing possible effects of salt-sensitivity on vitamin D status of humans.

## Background

The Dahl salt-sensitive (S) rat is a widely studied model of salt-induced hypertension [1,2]. The Dahl S rat, but not the Dahl salt-resistant rat (R), develops hypertension, accompanied by hypovitaminosis D, when fed a high salt diet [3-5]. Plasma concentration of 25-hydroxyvitamin D (25-OHD), the liver metabolite of vitamin D, is a marker of vitamin D status. Plasma 25-OHD concentrations are similar in young S and R rats when the rats are fed a low salt diet (0.3% sodium chloride). High salt intake, however, causes significant decreases in plasma 25-OHD concentrations of S rats, but not R rats [3,5-7]. Blood pressure was shown to be directly correlated and plasma 25-OHD concentration inversely correlated with the number of days that young S rats were fed an 8% salt diet [3,6]. We demonstrated that Dahl S rats lose protein-bound vitamin D metabolites into urine and that this loss is markedly accelerated during high salt intake [8]. High salt intake, thus, creates a vitamin D deficiency state in Dahl S rats in the presence of standard amounts of dietary vitamin D [3,5-7].

Since S and R rats were originally bred from the Sprague Dawley rat (SD) [1], we tested the hypothesis that R and SD rats would be similar in their vitamin D endocrine system response to high salt intake and not develop hypovitaminosis D, as does the S rat.

## Methods

### Animals and diets

Male SD rats, Dahl S (SS/Jr) and R (SR/Jr) rats (130-150 g, 4-5 weeks old, twelve rats per type) were obtained from Harlan Sprague Dawley, Indianapolis, IN. All protocols involving animals were previously approved by the Morehouse School of Medicine Animal Care Committee. Guidelines followed were those of the Public Health Service and the revised animal welfare act as regulated by USDA. The rats were maintained as previously described [9]. They were housed in a room with 12 h light - dark cycles and, after one week of acclimation, six rats of each type were fed either a low (3 g/kg) or high (80 g/kg) salt diet (Harlan Teklad, Madison, WI) for three weeks. Diets, blood collection, and blood pressure measurements have been previously described [9].

### Plasma 25-hydroxyvitamin D concentration

Plasma 25-OHD concentration was measured as previously described [3]. Plasma samples were purified using a dichloromethane/methanol liquid-liquid extraction, followed by solid-phase extraction. Fraction 1 from the solid phase extraction was used to assay for 25-OHD by radioimmunoassay kits available at the time of the study from Amersham Corp. (Arlington Heights, IL).

### Statistics

A mean ± SEM was calculated for each group. Statistical significance (*P *< 0.05) was evaluated by the Mann Whitney test (SigmaStat, SPSS, Inc., Chicago, IL.).

## Results

**Figure 1:**
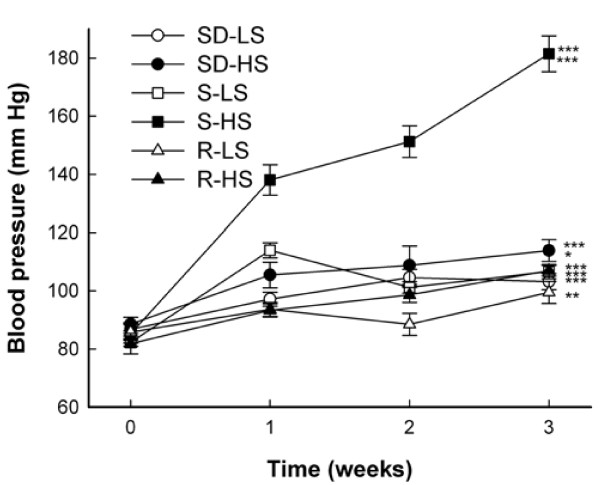
**High salt intake significantly increases the blood pressure of Sprague Dawley and Dahl salt-sensitive rats**. Sprague Dawley (SD) and Dahl salt-sensitive (S) and salt-resistant (R) rats were fed low (0.3%, LS) or high (8%, HS) salt diets for three weeks. Indirect blood pressure (systolic) was measured before initiation of the diets and weekly by tail cuff plethysmography. Values are means ± SEM, n = 6. ***P < 0.001, baseline vs. week 3 of all groups except R-LS (**P = 0.01). ***P < 0.001, S-HS vs. S-LS at week 3. *P < 0.05, SD-HS vs. SD-LS at week 3.

**Figure 2:**
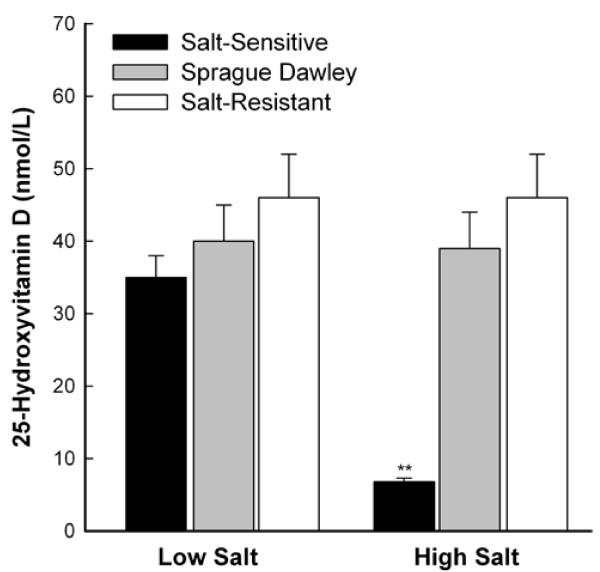
**Plasma 25-hydroxyvitamin D concentrations of Sprague Dawley and Dahl salt-sensitive and salt-resistant rats**. Sprague Dawley and Dahl salt-sensitive and salt-resistant rats were fed low (0.3%) or high (8%) salt diets for three weeks. Plasma samples were pre-purified by previously published methods [3] and 25-hydroxyvitamin D was assayed using a commercial kit (Amersham Corp., Arlington Heights, IL). Values are means ± SEM, n = 6. **P = 0.002, high salt intake vs. low salt intake, salt-sensitive rats.

Blood pressure significantly increased from baseline to week 3 among all groups (Figure 1). S and SD rats, but not R rats, exhibited significantly higher mean blood pressures at week 3 of high salt intake than at week 3 of low salt intake. The blood pressures plotted (Figure 1) are means for six rats/dietary group (set 1) of 12 S and 12 R rats/dietary group in a previous report [9]. Set 1 of that study included 12 SD rats, 6/dietary group. Mean plasma 25-OHD concentrations of SD, R, and S rats were similar at week 3 of low salt intake (Figure 2). Mean plasma 25-OHD concentrations of SD and R rats were similar at week 3 of high and low salt intake, whereas mean plasma 25-OHD concentration of S rats at week 3 of high salt intake was only 20% of that at week 3 of low salt intake.

## Discussion

The blood pressure of SD rats increased from baseline to week 3 during both high and low salt intake, and the mean blood pressure at week 3 of high salt intake was significantly higher than that at week 3 of low salt intake. Mean blood pressure of SD rats at week 3 of high salt intake was 114 ± 4 mm Hg, compared with means of 107 ± 2 mm Hg for R rats and 182 ± 6 mm Hg for S rats. Plasma 25-OHD concentrations of SD and R rats were unaffected by high salt intake, whereas mean plasma 25-OHD concentration of S rats at week 3 of high salt intake was decreased to 20% of that at week 3 of low salt intake. These data indicate that the vitamin D endocrine system response of SD rats to a high salt load (8%) for three weeks is more similar to that of R rats than to that of S rats.

Dahl S rats, but not Dahl R rats, are insulin-resistant [10-12]. Ogihara et al. [13] have demonstrated that a high salt diet induces insulin resistance in both S and SD rats. It has thus been suggested that the SD rat is essentially the same as the Dahl S strain [14]. Channa et al. [12] have suggested that insulin resistance and hypertension may be inherited as separate traits. In this study, the blood pressure response of SD rats to high salt intake was between that of R and S rats. This and other studies suggest that SD rats are similar to S rats in the induction of insulin resistance by high salt intake [13,14], but similar to R rats in the vitamin D endocrine response to high salt intake. The S rat, thus, appears to be a more appropriate model than the SD rat for assessing possible effects of salt-sensitivity on vitamin D status of humans.

## Competing interests

The authors declare that they have no competing interests.

## Authors' contributions

MT-P participated in the conception and design of the study and the vitamin D metabolite analysis and drafted the manuscript. TKT carried out the vitamin D metabolite analysis. NLE participated in the conception and design of the study. MAB participated in the conception, design, and coordination of the study and in the blood pressure measurements. All authors read and approved the final manuscript.
